# Integrated host and viral transcriptome analyses reveal pathology and inflammatory response mechanisms to ALV-J injection in SPF chickens

**DOI:** 10.1038/srep46156

**Published:** 2017-04-12

**Authors:** Xi Lan, Yan Wang, Kai Tian, Fei Ye, Huadong Yin, Xiaoling Zhao, Hengyong Xu, Yong Huang, Haibo Liu, John C. F. Hsieh, Susan J. Lamont, Qing Zhu

**Affiliations:** 1 Farm Animal Genetic Resources Exploration and Innovation Key Laboratory of Sichuan Province, Sichuan Agricultural University, Chengdu Campus, 611130, Sichuan Province, China; 2 Department of Animal Science, Iowa State University, Ames, 50010, Iowa, USA; 3 College of Veterinary Medicine, Sichuan Agricultural University, Chengdu Campus, Sichuan Province, China

## Abstract

Avian leukosis virus (ALV) is detrimental to poultry health and causes substantial economic losses from mortality and decreased performance. Because tumorigenesis is a complex mechanism, the regulatory architecture of the immune system is likely to include the added dimensions of modulation by miRNAs and long-noncoding RNA (lncRNA). To characterize the response to ALV challenge, we developed a novel methodology that combines four datasets: mRNA expression and the associated regulatory factors of miRNA and lncRNA, and ALV gene expression. Specific Pathogen-Free (SPF) layer chickens were infected with ALV-J or maintained as non-injected controls. Spleen samples were collected at 40 days post injection (dpi), and sequenced. There were 864 genes, 7 miRNAs and 17 lncRNAs differentially expressed between infected and non-infected birds. The combined analysis of the 4 RNA expression datasets revealed that ALV infection is detected by pattern-recognition receptors (TLR9 and TLR3) leading to a type-I IFN mediated innate immune response that is modulated by IRF7 and IRF1. Co-expression network analysis of mRNA with miRNA, lncRNA and virus genes identified key elements within the complex networks utilized during ALV response. The integration of information from the host transcriptomic, epigenetic and virus response also has the potential to provide deeper insights into other host-pathogen interactions.

Improvements in poultry health may serve to improve human health and food availability, and also support income generation^1^. Avian leukosis/sarcoma viruses, which belong to *Alpharetrovirus* genus of family *Retroviridae*, cause neoplastic diseases and decrease reproductive performance in the poultry industry worldwide^2^. Among the six subgroups of ALVs, avian leukosis virus subgroup J (ALV-J) infection causes significant economic losses due to increased mortality, tumor production, decreased production, and high costs for eradication. The subclinical disease syndrome is characterized by depressed egg production in the absence of tumor formation. The pathogenic mechanisms of host immunosuppression induced by ALV-J are poorly understood.

In addition to inhibition of growth and reproduction, ALV-J infection damages immune organs, including the spleen, bursa and thymus^3^. Infection with ALV-J causes changes in the bursal transcriptome that are involved in binding, biological regulation, metabolic processes, immune system processes and genes involved in tumourigenesis^4^. The spleen, an important immune organ in vertebrates, is the site at which the virus reaches its primary targets of B cells and CD4+ T cells during the infection process^5^. Li *et al*. reported that ALV-J infection causes hundreds of genes and miRNAs in the spleen to be differentially expressed^6^. Thus, characterizing the spleen’s genetic regulation during ALV-J infection will help to clarify the interaction between host genomics and the virus.

Recently, next generation sequencing (NGS), with highly sensitive measurements of whole transcriptomes, has become a significant technological advance in biological sciences^7^. Many studies with NGS technology have revealed a variety of biological mechanisms by monitoring the whole genome regulation under specific conditions^8^,^9^,^10^. Genome wide transcriptomic analysis through RNA sequencing (RNA-seq) is a promising tool to discover global gene expression change profiles, providing insights that are not limited to protein-coding RNA but also critical non-coding RNA^11^. Fifty percent or more of the, including short noncoding RNAs (Such as miRNAs) and long noncoding RNAs (lncRNAs), does not encode proteins but can play an important regulatory role responsible for the modulation of gene expression, especially in disease processes^12^. Also, host and viral RNA transcripts can be assayed simultaneously, allowing identification of host-pathogen interactions.

Hundreds of miRNA genes have been found in a large diversity of animals, and miRNAs are thought to have significant roles in developmental timing, cell death, cell proliferation, signaling pathway, apoptosis and metabolism, myogenesis and cardiogenesis^13^. Emerging evidence suggests a direct link between miRNAs and disease, where differential miRNA expression signatures are associated with various types of cancer in mammals^14^,^15^,^16^,^17^. The miRNA expression patterns in response to Marek’s Disease virus or ALV-J infection have been identified by microarray^18^,^19^,^20^,^21^, and miRNA-23b was confirmed to target IRF1 to promote ALV-J replication^6^. Thus, miRNAs are not only associated with tumorigenesis, but may also play a critical role in the interaction between host and pathogen.

Previous studies suggest that lncRNAs may represent a major component for the regulation of the eukaryotic genome^22^. A large range of functions have been attributed to lncRNAs, including modulation of apoptosis and invasion^23^, reprogramming of induced pluripotent stem cells^24^, and as markers of cell fate^25^ and parental imprinting^26^. The lncRNAs are increasingly being implicated in mediating host response and immune function, suggesting an elaborate network of regulatory interactions mediated through lncRNAs during infection^27^,^28^. Although studies have revealed the function of many noncoding RNAs, the specific role of lncRNAs in host-pathogen cross talk has not been previously explored in poultry^29^.

In our RNA-seq experiments, we measured changes in total mRNA, noncoding RNA and ALV RNA induced in the spleen of ALV-J infected chickens. To our knowledge, this is the first application of NGS technology to characterize host-ALV interactions in response to an ALV-J challenge. We directly measured ALV mRNA in infected spleen tissue, and also measured host expression of mRNA and noncoding RNA, including miRNA and lncRNA. This study not only increases knowledge about ALV-host interaction, but will also provide a framework for future studies of host-pathogen interactions.

## Results

### Chicken spleen and virus transcriptome

**Figure 1:**
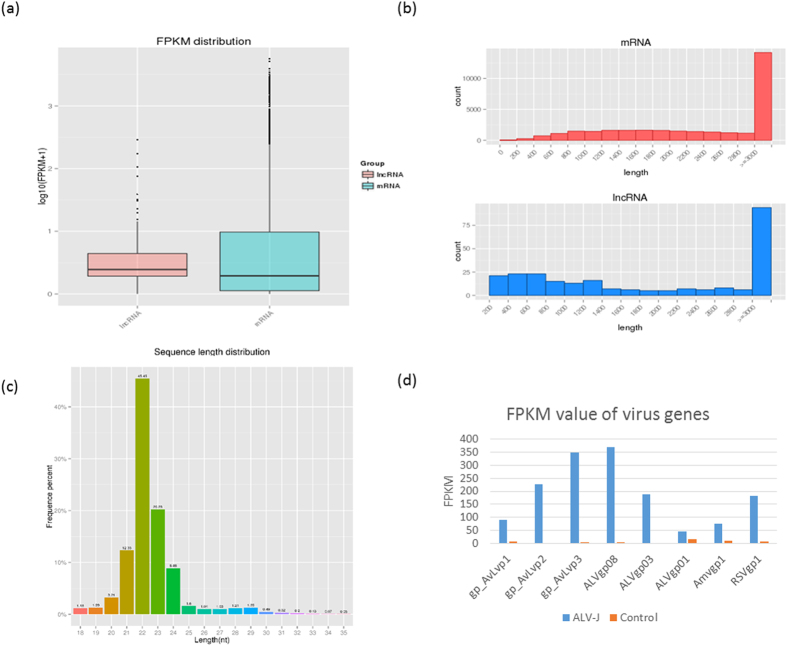
Basic information of RNA-seq data. (**a**) Box plot of Fragment Per Kilobase of transcript per Million mapped reads (FPKM) for lncRNA and mRNA. (**b**) Read length distribution for mRNA and lncRNA. (**c**) Read length distribution for miRNA. Most miRNA are in the 21–24 nt range. (**d**) FPKM value of ALV-J gene fragments: gp_AvLvp1 and ALVgp01 from *env*, gp_AvLvp2 from *gag*, gp_AvLvp3 and ALVgp08 from *pol*, and ALVgp03 from a trans-acting factor. Two other viral gene fragments were also included: Amvgp1 from avian myelocytomatosis virus and RSVgp1 from Rous sarcoma virus. Blue bar is the ALV-J treatment group and orange bar is the control group.

In total, approximately 843 million 100 bp paired-end reads were generated using the Illumina HiSeq 2000 for six (three infected, three control) spleen cDNA libraries. This yielded 84.3 gigabases of sequence in total, representing about 84.3-fold coverage of the chicken genome and provided on average 140 million (range, 131–151 million) reads per sample. Approximately 79.62% of the reads were uniquely mapped to the *Gallus gallus* 4 genome (Supplemental Table S1). Expression of 16,833 annotated genes (98.39% of the 17,108 gene set) and 254 lncRNA were detected in at least one individual. The boxplot of FPKM and histogram of length for both mRNA and lncRNA are illustrated in Fig. 1a and b.

After quality filtering, 64 million out of 67 million raw miRNA reads were used for downstream analysis. Of these reads, 90.70% were 19–24 nt long (Fig. 1c), 55.38% of the reads mapped uniquely to the *Gallus gallus* 4 genome and 70.98% of the reads were assigned to miRNA (Supplemental Table S1). Over 18.0% (110/610) of the miRNAs expressed over 100 reads per million (RPM), and, therefore, were likely to be functional.

A large number of ALV sequencing reads were mapped to the ALV genome: 791,574 reads (0.2% of the total reads). Eight virus genes were detected to be expressed in the ALV challenged group, with ALVgp08 having the greatest sequencing depth at 368 FPKM and ALVgp01 having the least sequencing depth at 44 FPKM. In comparison, the control group had negligible expression levels with the highest detected virus gene. The virus gene expression levels are shown in Fig. 1d.

### Principle Component Analysis and differential expression analysis

**Figure 2:**
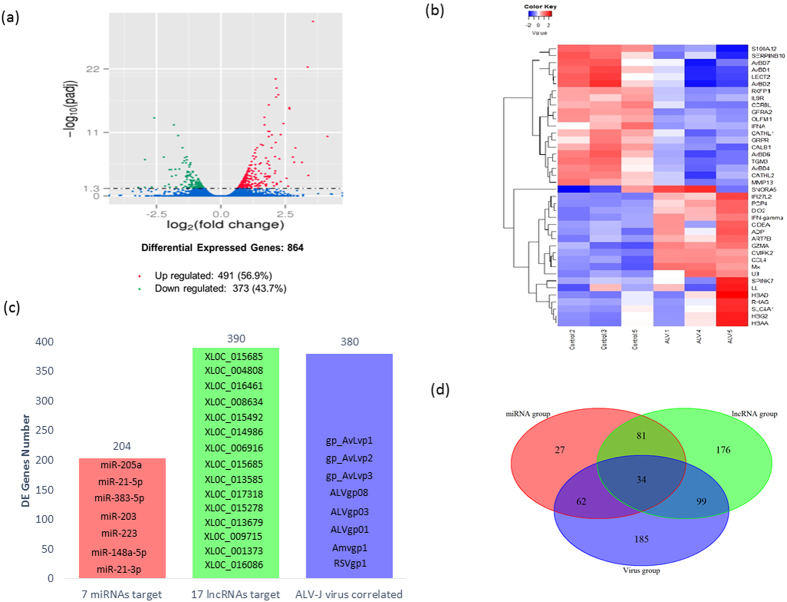
Differentially expressed genes (DEGs) results. (**a**) Volcanic plot of DEGs. Red dot represents significantly up-regulated DEGs (*n* = 491), green dot represents significantly down-regulated genes, and blue dots represents non-differentially expressed genes. (**b**) Heatmap of the top 20 DEGs (*n* = 373) based on absolute fold changes. (**c**) Number of targeted DEGs by miRNAs, lncRNAs and virus genes. Red bar represents 204 DEGs targeted by 7 differentially expressed miRNAs, green bar represents 390 DEGs targeted by 17 differentially expressed lncRNAs, and blue bar represents 380 DEGs correlated with the 8 viral gene fragments. (**d**) Venn diagram showing the overlap of the three targeted DEGs groups from (**c**). Red circle is the miRNA group, green circle is the lncRNA group, and blue circle is the virus group.

The PCA shows tight clustering within the ALV treatment and within the control group for mRNA, miRNA, and lncRNA expression (Supplemental Figure S2a–c, respectively). A total of 864 genes were identified as differentially expressed (DE) in response to ALV at 40 dpi with 56.9% of the genes up-regulated (Fig. 2a). The top 20 up and down-regulated genes based on fold change (FC) ranged from 10- to 17-FC (Fig. 2b). Up-regulated genes included IFN-related genes (IFN-α, Mx, IFI27L2), chemokine (CCL4) and cell death activator (CIDEA, Supplemental Table S3). Seven of the top 20 down-regulated genes were in the host defense peptide (HDP) gene family (Supplemental Table S3). Seven DE miRNAs predicted 260 targeted DE genes (“miRNA group”) and 14 DE lncRNAs predicted 390 target DE genes (“lncRNA group”, Fig. 2c). Additionally, without multiple testing correction, a total of 48 miRNAs were found to be differentially expressed (p < 0.05) between the ALV-injected group and the control group, with 25 down-regulated miRNAs and 23 up-regulated miRNAs. The expression levels of 8 ALV genes were correlated with 380 DE genes (“virus group”, Fig. 2c). The Venn diagram of DE genes from the miRNA, lncRNA and virus groups showed 34 shared DE genes, including CCNB2, SATB1, GCH1and CALB1 (Fig. 2d).

### Ingenuity Pathway Analysis

**Figure 3:**
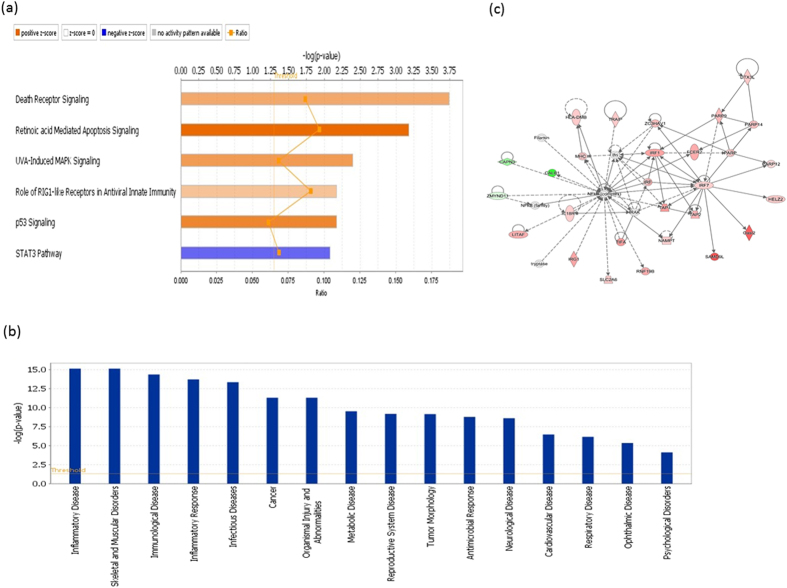
IPA Functional analysis of DEGs correlated with viral genes. (**a**) Top canonical pathways. More orange indicates more activation while more blue indicates more inhibition of pathways. (**b**) Downstream disease and biological pathways. (**c**) Predicted network showing numerous immune related genes around 3 central hubs (NF-κB, IRF1 and IRF7) that are associated with pathogen recognition process. Dotted lines are indirect interactions and solid lines are direct interactions. Genes in red are up-regulated DEGs, genes in green are down-regulated DEGs, and genes in grey are predicted by IPA.

Canonical pathway analysis from the virus-injected group showed 6 significant pathways (|z-score| >2) that were all immunity-related, with 5 of those pathways activated and only the STAT3 pathway inhibited (Fig. 3a). There were 65 activated upstream regulators predicted by IPA, and nearly all of the top 20 regulators were related to innate immunity, including IFNs (7 genes), IRFs (4 genes) and TLRs (2 genes, Supplemental Table S4). Additionally, 16 downstream functions were predicted; inflammatory and immune response disease was the top predicted function, and metabolic and reproductive system diseases were also significant (Fig. 3b). Additionally, a novel network was predicted by IPA with three central hubs (NF-κB, IRF1 and IRF7) that are associated with the pathogen recognition process (Fig. 3c).

The IPA functional analysis of the miRNA group identified 204 out of 260 genes involved with cancer functions. Canonical pathway analysis of the miRNA group included all pathways found from the canonical pathway analysis of the viral group, and the additional activated pathways of death receptor signaling, retinoic acid mediated apoptosis and UVA-induced MAPK biosynthesis I, and inhibited pathway of cAMP mediated signaling. The upstream analysis also predicted the activation of 27 immune related genes, including IRF1, IL5, IFNG and STAT1 (Supplemental Table S5). Also, the IPA functional analysis predicted, 254 out of 390 genes from the lncRNA group were involved in cancer diseases. Canonical pathway analysis of the lncRNA group included all pathways found from the canonical pathway analysis of the viral group, with the additional inhibited pathway of glioma signaling.

**Figure 4:**
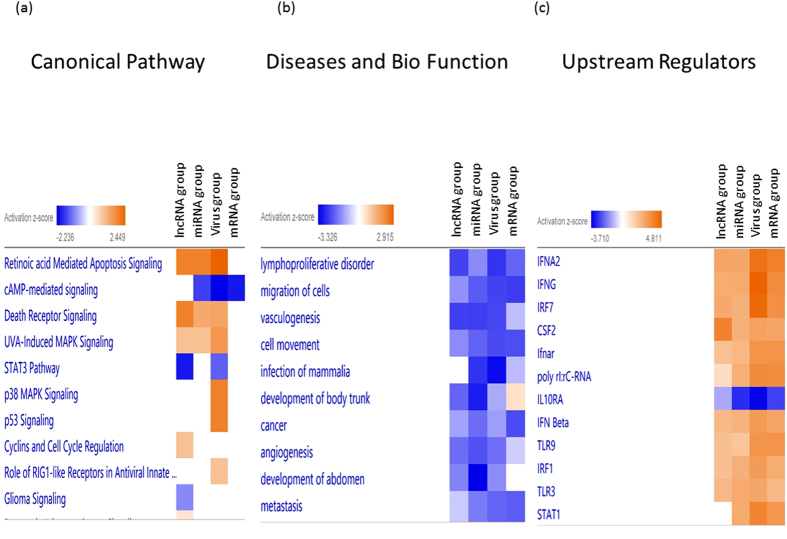
Heatmap comparing IPA functional analyses of the 4 groups. From left to right: lncRNA group, miRNA group, virus group and mRNA group. (**a**) Top canonical pathways. (**b**) Diseases and biological pathways. (**c**) Predicted upstream regulators.

IPA was also used to gain a global perspective on the genes that were modulated in common among the different types of genetic elements. We combined the DE gene lists from all 4 datasets (mRNA, miRNA, lncRNA, and virus) to create several heatmaps. Several important significant canonical pathways were shared among all the datasets. Two of the top three pathways “Retinoic acid Mediated Apoptosis Signaling” and “Death Receptor Signaling” were activated, while “cAMP-mediated signaling” was inhibited (Fig. 4a). The absolute z-score was the lowest in the mRNA group for the canonical pathway analysis. Interestingly, almost all of the predicted downstream diseases and functions were inhibited including “lymphoproliferative disorder”, “migration of cells” and “vasculogenesis” (Fig. 4b). The analysis of, upstream regulators showed good agreement with results from the four datasets (Fig. 4c). Most of the top activated upstream molecules are related to innate immunity, with high enrichment in the pattern-recognition receptors pathway and immune response, including IFNA2, IFNG, IRF7, CSF2, IFN beta, TLR9, IRF1, TLR3 and STAT1.

### Co-expression network analysis among mRNA, miRNA, lncRNA

**Figure 5:**
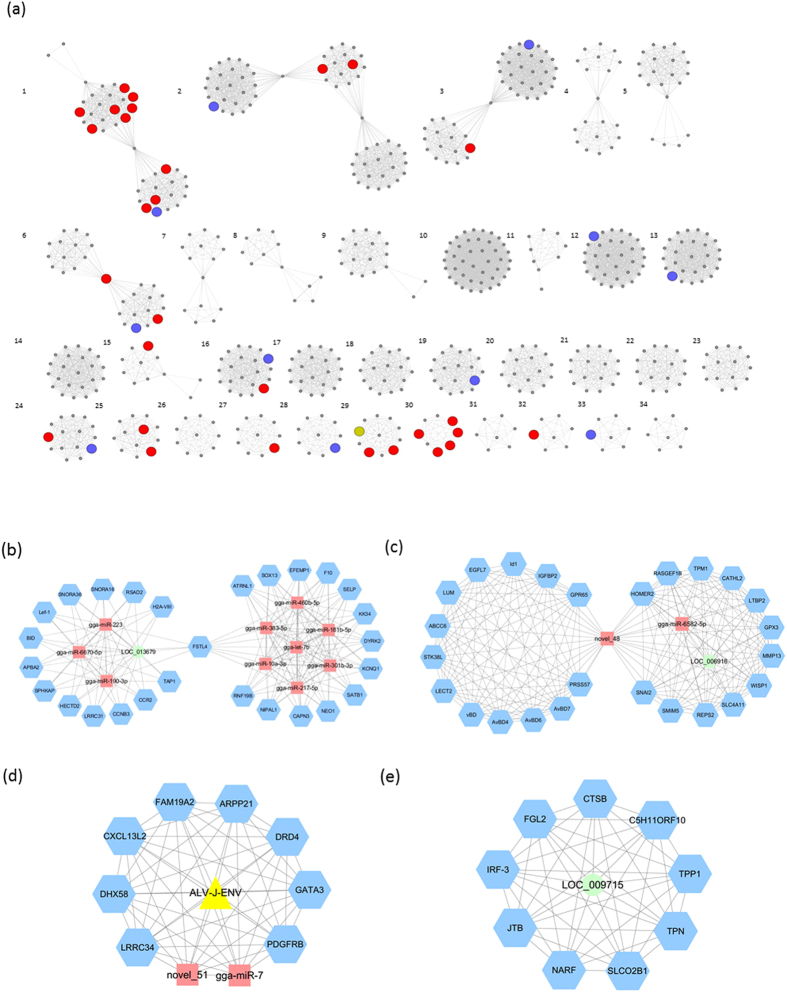
Co-expression network of mRNA DEGs, DE miRNAs, DE lncRNAs and expressed viral genes. (**a**) Network analysis identified 34 networks involving 7 or more nodes. (**b**) Network 1 contained 10 miRNAs, one lncRNA and 27 protein-coding genes. (**c**) Network 6 has a novel miRNA (novel_48) as the central hub that combines 2 modules of protein-coding genes. (**d**) Network 28 has an ALV gene (*env*) directly interacting with 2 miRNAs (miR-7 and novel_51) and 8 protein-encoding genes. (**e**) Network 29 has an lncRNA (LOC_009715) as the central regulator of 9 protein-encoding genes.

To investigate stimulus-specific genes associated with the corresponding regulatory effectors, WGCNA was used to determine specific gene-expression correlations for all DE protein-coding genes, DE miRNAs, DE lncRNAs and DE virus genes. We identified 34 distinct co-expression networks containing 5 to 62 nodes (Fig. 5a). There were 4 networks (1, 6, 28 and 29) that contained nodes related to ALV-J stimulation and immune response.

Network 1 contained 10 miRNAs, one lncRNA and 27 protein-coding genes with FSTL4 as the central hub (Fig. 5b). Nineteen of the 27 protein-coding genes were related to variety of diseases and cancers. One module in the network contained 3 miRNAs (miR-223, miR-6670-5p and miR-190-3p) and one lncRNA (LOC_013679) interacting with 14 protein-coding genes, including Lef1 and TAP1. The other seven miRNAs (miR-460B-5P, miR-383-5p, miR-181b-5p, gga-let-7b, miR-301b-3p, miR-10a-3p and miR-217-5p) created the other half of the network, interacting with 13 other protein-coding genes that are related to the immune system, hemarthrosis, arteriosclerosis obliterans, apoptosis, survival caspase cascade and muscular dystrophy.

A novel miRNA (novel_48) was the central hub that combined 2 modules of protein-coding genes in network 6 (Fig. 5c). All of the protein-encoding genes in this network were related to immune response to diseases. One of the modules contained the HDPs genes family (AvBD1, AvBD4, AvBD6 and AvBD7), and the other module included 10 of the 12 protein-encoding genes related to diseases function, such as RASGEF1B with a functional role in releasing of GDP to activate RAS signaling.

In one of the two smaller networks identified, network 28, an ALV gene (*env*) had direct interaction with 2 miRNAs (miR-7 and novel_51) and 8 protein-encoding genes (Fig. 5d). One gene, DHX58, is part of the RIG-I/MDA5 mediated induction of IFN. The lncRNA LOC_009715 is a central regulator in network 29 with 9 protein-encoding genes (Fig. 5e). IRF3, which is a critical gene in innate immunity mediated by IFN, was identified in this network to directly interact with the lncRNA. Other protein-encoding genes (FGL2, CTSB, TPP1, SLCO2B1, NARF and JTB) have disease related functions, such as viral hepatitis and ileum cancer.

### Validation of RNA-seq data by real-time RT-PCR

Based on the immune response, 20 up-regulated and 12 down-regulated genes were selected for qPCR analysis (Supplemental Table S6). All samples used in the RNA-seq analysis, plus samples from an additional 4 chickens (two of each treatment group) were included in the qPCR analysis, resulting in 5 samples per treatment. The qPCR and RNA-seq results were highly correlated (R^2^ = 0.95, Supplemental Figure S7), indicating a close agreement between the two methods. The data demonstrated that real-time RT-PCR results were consistent with those of the RNA-seq, although several-fold differences were observed in results between the two analytical methods because of intrinsic differences between the techniques.

## Discussion

ALV-J in egg-type chickens causes immunosuppression and leads to decreases in body weight, egg production, fertility and egg hatchability, resulting in major economic losses^30^. To provide a comprehensive view of the transcriptome level changes that occur within the spleen of chickens that have been infected with ALV-J, whole transcriptome analysis was used to elucidate the candidate genes’ function and their regulatory effectors, and the host-pathogen interaction in host immunity and neoplastic etiology. In total, 864 genes, 7 miRNAs and 17 lncRNAs were differentially expressed in the spleen of the ALV-infected chickens, reflecting immune response to the viral challenge.

Host defense peptides, which include defensins (AvBD), cathelicidins (CATH-) and liver expressed antimicrobial peptide-2(LEAP2), have antimicrobial activity against many pathogens, including Gram-positive and -negative bacteria, fungi and even enveloped viruses^31^. The killing mechanisms of beta-defensins include positively charged residues interacting with negatively charged membrane components, after which hydrophobic residues insert into the membrane, disrupting it and killing the pathogen^31^. Few studies, however, have investigated defensin expression in response to viral infections in poultry. The AvBD7 reported as upregulated in liver and bone marrow in response to duck hepatitis virus infection^32^. The AvBD4 expression changed after vaccination with fowlpox virus containing the hemagglutinin gene of avian influenza^33^. Moreover, CATH-2 also has been reported as an important gene that is differentially expressed in the chicken bursal transcriptome in response to ALV-J infection^34^. In the current study, ALV-J strongly suppressed the expression of defensins (AvBD1, AvBD2, AvBD4, AvBD6, AvBD7) and cathelicidins (CATH-1 and CATH-2). These result clearly reveal one mechanism, down-regulation of the host defense peptide system, by which ALV suppresses immune function in the host and causes it to become more susceptible to other diseases.

In addition to anti-pathogenic functions, HDPs also have been shown to exhibit diverse roles in immunomodulatory effects^31^,^35^. The HDPs can not only induce pro-inflammatory cytokine expression by binding directly to chemokine receptors, but also can induce an anti-inflammatory response through blocking LPS-induced inflammation^31^. AvBD13 was reported to induce the expression of CD88, CD86 and NF-κB, and the secretion of IL-12 and IFN-gamma in mouse monocytes^36^. Moreover, an *in vivo* study found that AvBD1 increased antibody titers and CD3+, CD4+ and CD8+ T-cell proliferation^37^. A novel co-expression network identified four AvBD genes interacting with 9 other immune-related genes that are associated with diseases. Although HDPs were significantly inhibited by ALV infection in the current study, their specific roles in immunomodulatory activities still remain to be identified. The HDPs found in this study might be interesting genes to further study to determine how ALV suppresses the immune response in chicken.

Genomic sequencing approaches have not only enabled the identification of roles for common functional genes involved in pathways related to disease pathogenesis, but can also be utilized to reveal noncoding RNAs with regulatory effects. Many prior studies have reported the critical role of miRNA in various aspects of the immune system^38^,^39^,^40^. In the current study, 7 DE miRNAs were identified between the ALV-J challenged and control groups. Six of these miRNA have been extensively studied in other organisms: (1) miR-205 has complex roles in tumor initiation and progression in multiple cancers^41^, (2) miR-21 has been identified as a candidate biomarker for multiple cancers^42^,^43^,^44^, (3) miR-383-5p has a role in dendritic cell maturation and macrophage activation^45^, (4) miR-148 has been associated with the HIV control^46^, (5) miR-203 has inhibiting effects on skeletal muscle cell proliferation and differentiation^47^ and (6) miR-223 has a mediator role in the development of inflammatory bowel disease^48^. Our study helped to expand the knowledge of the regulatory, functional and applied roles of these miRNAs to a species of scientific and economic importance.

In recent years, numerous reports have identified thousands of actively expressed lncRNA transcripts with distinct properties, especially in tumorigenesis. ALV-J induces a neoplastic disease. The current study is the first to identify DE lncRNAs through whole transcriptome sequencing after an ALV-J challenge. Only 17 lncRNAs out of 254 expressed lncRNAs were determined as DE. Because most lncRNA’s functions are still unknown, we predicted 390 target DE genes with the 17 DE lncRNAs. Among the lncRNA group, 254 genes were predicted to have involvement in cancer, suggesting that dysregulation of lncRNA expression is a crucial step during viral infection response in chicken. Further support for this hypothesis is provided by the co-expression network analysis result of LOC_009715 having a direct interaction with IRF3, which directly induces IFN type-I production for innate immune response^49^.

Host response to viral infections involves a complex set of regulatory processes. It has also been suggested that viruses utilize host miRNAs during interaction with the host. Our co-expression network analysis showed that ALV has highly correlated expression with two miRNAs, miR-7 and novel miRNA 51. Previously, miRNA-7 has been reported to modulate the expression of several oncogenes^50^,^51^,^52^. Analysis of the 380 DE genes from the virus group in IPA suggest that the mechanisms of the host response to pathogen include both recognition and defense against the pathogen, and also the activation of apoptosis and inflammatory pathways: 1) canonical pathway analysis identified p53 signaling, RIG-I and STAT3 pathway, 2) downstream functional analysis predicts a wide range of immune responses, and 3) network analysis found a network with 3 central nodes of NF-κB, IRF1 and IRF7, surrounded by several up-regulated genes that are involved in various cancers. Including the host response, therefore, in studies of viral gene expression will improve the understanding of neoplastic etiologies induced by ALV.

NGS technology enables a comprehensive monitoring of gene expression for both pathogen and host during their interaction. Combining datasets from the mRNA group, miRNA group, lncRNA group and virus group, the heatmap of IPA combined analyses gave a more comprehensive view into the host splenic transcriptome response to ALV-J infection. For example, by utilizing all 864 DE genes to perform the canonical pathway analysis the resulting pathways are less focused on viral responses, as illustrated by the inclusion of non-immune related DE genes. In contrast, the same analysis performed with the other 3 datasets showed much stronger signals for the relevant immune response, highlighting the power of combining the different data sources.

From the use of combined datasets in IPA, it is evident that the chicken innate immune system recognizes the ALV-J via the pathogen-associated pattern-recognition receptors, TLR9 and TLR3. After the recognition of ALV-J viral components, increased production of type-I IFNs (IFN-alpha and IFN-beta) for activation of target immune cells depended on activation of known upstream regulators (IRF7 and IRF1) and the inhibition of pro-inflammatory cytokine (IL10RA) in the STAT1-mediated signaling pathway^49^. This resulted in suppression of the proliferation and movement of cells, vasculogenesis, angiogenesis and metastasis. The analysis of combined datasets in IPA helped to elucidate a comprehensive picture of the activation process of the host immune system in response to ALV-J infection.

Infection initiates a dynamic cascade of events that culminates in altered gene expression patterns in both interacting organisms: pathogen and vertebrate host. These changes may lead to the adaptation and persistence of the pathogen or to its clearance from the host by immune response. The goal of co-expression network analysis is to identify groups of genes that are highly correlated in expression levels across multiple conditions. The genes in the same co-expression network often have related functions. In our study, we found two networks that combined lncRNA and miRNAs with several DE genes related to immune response. Also, the expression of ALV-J virus genes directly correlated with two miRNAs known to play a critical role in cancer response. An unbiased transcriptomic analysis of both host and pathogen can provide new insights into novel processes of the host-pathogen interaction by identifying new genes and pathways in the host cell in response to exposure of pathogen or pathogen-associated molecular pattern. In addition, functions of novel miRNAs and lncRNAs involved in the modulation of host genes expression under ALV-J interaction can also be identified.

## Methods

### Chicken Challenge and Tissue Collection

Seventy embryos of SPF (Specific Pathogen-Free) white Leghorn egg-type chicken (Merial, Beijing, China) were divided equally into two groups. After hatch, the chicks were maintained separately in two pathogen-free negative pressure isolators (Strong Star Equipment Technology Co, Qingdao, China) and given ultraviolet sterilized food and boiled-water *ad lib* for 40 days. At one day of age, ALV-challenged chicks were injected with 100 μl SCDY1 Avian leucosis virus subgroup J (ALV-J) (supplied by College of Veterinary Medicine, Sichuan Agricultural University, Sichuan, China) based on the TCID_50_ of this virus, while a control group was injected with 100 μl PBS per chick. Birds were euthanized at 3, 5, 7, 14, 24, 30, 40 days post injection (dpi) and the blood and immune organs were harvested. At 5 dpi, viral mRNA could be detected in infected chicks. All samples were tested for ALV-J by 3 methods: 1) PCR followed by sequencing of the viral DNA, 2) qPCR for the viral mRNA and 3) ELISA for ALV-J antibody. Based on an immune index, 40 dpi was determined to be near peak host response (Supplemental Figure S8) and therefore this age was used for the current study. The phenotype of infected tissues (including appearance, viability, and the amount of viral mRNA and protein) was used to guide the selection of samples for RNA-seq. Six spleens from 40 dpi (3 control and 3 ALV-J challenged) were used for RNA-seq. Spleen samples from 4 additional chickens (2 control and 2 challenged) were included in the qPCR validation of the RNA-seq results. All animal care and experimental procedures were reviewed and approved by the Animal Care and Use Committee (#YYS130125) of Animal Care Advisory at Sichuan Agricultural University. This study was carried out in strict accordance with the Regulations for the Administration of Affairs Concerning Experimental Animals of the State Council of the People’s Republic of China.

### RNA isolation, library construction and sequencing

Total RNA was isolated from the RNAlater-preserved spleen samples with miRNeasy Mini kit (QIAGEN, Gemany) in accordance to the manufacturer’s protocol. RNA was quantified using the NanoPhotometer^®^ spectrophotometer (Implen Inc., CA, USA). The RNA concentration was measured using Qubit^®^ RNA Assay Kit in Qubit^®^ 2.0 Flurometer (Life Technologies, CA, USA), and RNA integrity assessed using the RNA Nano 6000 Assay Kit in Bioanalyzer 2100 (Agilent Technologies, CA, USA). All the samples had a high RNA Integrity Number (RIN) score (>9.4). A total of 3 μg of RNA per sample was submitted to the Novogene Bioinformatics Technology Co. Ltd (Beijing, China) for library generation and sequencing. The libraries were sequenced using Illumina HiSeq 2000 platform with 6 lanes (one per sample), and 100 bp paired-end reads were generated.

### Sequence reads qualification, mapping, assembly and annotation

Raw read quality was assessed using the FastQC suite version 0.10.1 (http://www.bioinformatics.babraham.ac.uk/projects/fastqc/). Raw reads were processed with custom perl scripts to remove reads containing adapter, reads containing poly-N and low quality reads. All the downstream analyses were based on the clean data with high quality based on a rerun of FastQC. The RNA-seq reads were aligned using TopHat v2.0.9 (http://tophat.cbcb.umd.edu/), mapped to the reference genome based on the NCBI *Gallus gallus* Build 4.0 (Ensemble V81), and chicken virus database, the indices for which were built by Bowtie v2.0.6 (http://bowtie-bio.sourceforge.net/). HTSeq v0.6.1 (http://www-huber.embl.de/HTSeq/)^53^ was used to count the read numbers mapped to each gene. Supplemental Table S1 details the quality filtering and mapping steps.

### PCA and Testing for Differential Expression

DESeq2 (http://www.bioconductor.org/)^54^ was used to perform PCA. The RPKM, FPKM and RPM were used to analyze mRNA, lncRNA and miRNA expression level, respectively, for the PCA. Differential expression analysis between challenge and control groups was performed by edgeR version 1.10.1 (http://www.bioconductor.org/). EdgeR^55^ provides statistical routines for determining differential expression in RNA-seq gene expression data using a generalized linear model based on the negative binomial distribution. The resulting p-value was adjusted using the Benjamini and Hochberg approach for controlling the false discovery rate (FDR)^56^. Genes and lncRNAs with an FDR < 0.05 and fold change >1 found by EdgeR were declared to be DE genes and DE lncRNAs. An FDR < 0.05 was used as the threshold to identify the DE miRNAs. Additionally, *p < 0.05* was used to increase the number of significant miRNAs for a more focused co-expression analysis. To identify which DE genes were common among different predicted groups and which were characteristic only for a particular target group, Venn diagrams were used. The volcano plots of genes were generated in R, with genes highlighted in blue that had a FDR < 0.05. Moreover, the 40 genes with the largest log2 fold change between the ALV-J challenged group and control group were further displayed in heatmap, generated in R using the gplots package^57^.

### Identification of miRNA and predicted the target genes

The small RNA reads produced by Illumina HiSeq 2000 were subjected to several filtering processes: (1) filter out low quality reads, (2) trim 3′ adaptor sequence by a modified dynamic programming algorithm, (3) remove reads with 5′ adaptor contaminants, containing ploy-N and (4) retain only short trimmed reads of sizes from 18 to 30 nt. The Q20, Q30 and GC-content of the raw data were calculated. The small RNA tag were mapped to reference sequence by Bowtie to measure the expression (http://bowtie-bio.sourceforge.net/index.shtml)^58^. To annotate and classify small RNAs into different categories and identify novel miRNA candidates, the following analyses were performed: (1) exclude small RNA reads that might be from protein-coding genes and known noncoding RNAs by comparing with known noncoding RNAs (only considering rRNA, tRNA, snRNA, and snoRNA), (2) small tags mapped to RepeatMasker, Rfam database or from the specified species itself were excluded. After excluding small RNA reads in the preceding categories, the rest were used to predict novel miRNA by both miREvo and mirdeep2, which identifies miRNA candidates according to the canonical hairpin structure and sequencing data^59^,^60^. The identified miRNAs that were absent in the miRBase database (http://www.mirbase.org/, release 13.0) were regarded as novel miRNAs^61^. Predicting the target genes of miRNA was performed by psRobot_tar in miRanda^62^.

### Identification of lncRNA and predicted the target genes

Four tools were used to filter out predicted transcripts with coding potential: Coding-Non-Coding-Index^63^; Coding Potential Calculator^64^; Pfam-scan^65^ and phylogenetic codon substitution frequency^66^. All the software were used with default parameters, and those without coding potential were classified as candidate lncRNAs. There are two main ways for lncRNAs to modulate protein coding genes: (1) *cis* role in which lncRNA act on neighboring target genes (10k upstream and downstream), and (2) *trans* role in which lncRNA identify target coding gene by the mRNA expression level. We calculated the correlation between lncRNAs and coding genes with custom scripts to search for correlated expression.

### IPA Analysis and Gene Co-Expression Network Analysis

Ingenuity Pathway Analysis (IPA) software (QIAGEN Inc., CA, USA) was used to discover relevant modulated pathways and transcriptional networks according to Fisher’s exact test enrichment statistics and Benjamini-Hochberg corrected p-value calculations. Four gene lists sets were input for the IPA analysis, including mRNA DE genes, miRNA targeted DE genes, lncRNA targeted DE genes and viral co-expressed DE genes. Genes from the data set with more than 1-fold up or downregulation and an FDR of 0.05 that were associated with biological functions in the Ingenuity Knowledge Base were included in the IPA analysis, including predicting the upstream biological regulators and possible downstream effects on cellular and organismal biology. Gene networks were also constructed by IPA^67^.

To systematically explore the interactions and determine the clusters of co-regulated genes and their putative regulators, co-expression networks were created from mRNA, miRNA, lncRNA, and viral RNA expression levels. To reduce the number of candidate targets, only the DE genes were selected to construct the co-expression network. Weighted gene co-expression network analysis (WGCNA) was used to explore the interaction of mRNA, miRNA, lncRNA and virus genes^68^. Only DE genes (FDR < 0.05), DE miRNA (p < 0.05), DE lncRNA (p < 0.05) and virus genes were included for WGCNA analysis. Co-expression network analysis by WGCNA was based on Spearman correlation with cutoff set at r^2^ > 0.975. Cytoscape (http://www.cytoscape.org/) was used to visualize the predicted network^69^. The flowchart for this portion of the analysis can be found in Supplemental Figure S9.

### Quantitative PCR (qPCR) and statistical analysis

Twenty-six genes were selected to confirm RNA-seq results. GAPDH and β-actin were the housekeeping genes used to normalize the starting concentration of RNA. The forward and reverse primers are listed in Supplemental Table S6. Total RNA was isolated according to previously described procedures. Quantitative PCR was performed using PrimeScript™ RT reagent Kit and SYBR^®^ Premix Ex Taq™II (TaKaRa, Dalian, China). All reactions were run in triplicates. The amplicons were verified for specificity and reaction quality using the melting curves of the reactions. The cycle threshold (Ct) line was adjusted to fit the standard curve with an acceptable r^2^ value, 0.96-1.00. The mRNA expression levels as mean adjusted Ct values of each triplicate sample were analyzed on combined data between control and challenge groups for each gene separately. Student’s t test of SAS software was used to determine significant differences (p < 0.05) between ALV challenge and control groups. Additionally, the Pearson correlation coefficient was used to determine the degree of correlation for each selected gene between the qPCR and RNA-seq. The fold change value of each gene’s expression comparing the challenged group and control group was used.

### Data deposition

RNA-seq data sets generated from this study are available in the Array Express database (www.ebi.ac.uk/arrayexpress) with the accession number E-MTAB-5135.

## Additional Information

**How to cite this article**: Lan, X. *et al*. Integrated host and viral transcriptome analyses reveal pathology and inflammatory response mechanisms to ALV-J injection in SPF chickens. *Sci. Rep.* **7**, 46156; doi: 10.1038/srep46156 (2017).

**Publisher's note:** Springer Nature remains neutral with regard to jurisdictional claims in published maps and institutional affiliations.

## Supplementary Material

Supplementary Information

Supplementary Table 4 and 5
