# Supplementary material for: Integrated host and viral transcriptome analyses reveal pathology and inflammatory response mechanisms to ALV-J injection in SPF chickens
Source: Sci Rep. 2017 Apr 12;7:46156. doi: 10.1038/srep46156 (PMC5388866; doi:10.1038/srep46156)

Online Supplemental

**Integrated host and viral transcriptome analyses reveal pathology and inflammatory response mechanisms to ALV-J injection in SPF chicken**

**Xi Lan^1, 3^, Yan Wang^1^, Kai Tian^1^, Fei Ye^1^, Hua-dong Yin^1^, Xiao-ling Zhao^1^, Heng-yong Xu^1^, Yong Huang^2^, Hai-bo Liu^3^, John C.F. Hsieh^3^, Susan J. Lamont^3^, Qing Zhu^1*^**

1. Farm Animal Genetic Resources Exploration and Innovation Key Laboratory of Sichuan Province, Sichuan Agricultural University, Chengdu Campus, 611130, Sichuan Province, China

2. College of Veterinary Medicine, Sichuan Agricultural University, Chengdu Campus, Sichuan Province, 611130, China

3. Department of Animal Science, Iowa State University, Ames, 50010, Iowa, USA

**Correspondence Author:** Qing Zhu, Institute of Animal Genetics and Breeding, Farm Animal Genetic Resources Exploration and Innovation Key Laboratory of Sichuan Province, Sichuan Agricultural University, Ya'an, 625014, P.R.China.

E-mail: zhuqing5959@163.com;

Telephone number: +86-835-2882006;

Fax number: +86-835-2883153.

Supplemental Table S1. Basic information on sequencing output and processing

| Sample | Total raw reads | Total raw base pair | Mapping reads percentage | Total sRNA reads | Mapped sRNA reads | Mapped sRNA percentage |
| --- | --- | --- | --- | --- | --- | --- |
| Control 2 | 134,450,394 | 13.44G | 80.58% | 9,778,277 | 5,053,281 | 51.68% |
| Control 3 | 139,485,544 | 13.94G | 80.18% | 10,675,575 | 6,441,361 | 60.34% |
| Control 5 | 125,384,158 | 12.53G | 79.50% | 10,720,983 | 5,504,270 | 51.34% |
| ALV1 | 128,652,822 | 12.86G | 79.40% | 10,745,581 | 5,707,063 | 53.11% |
| ALV4 | 128,210,384 | 12.82G | 79.60% | 11,042,169 | 6,786,400 | 61.46% |
| ALV5 | 119,962,300 | 11.99G | 78.44% | 11,138,948 | 6,054,975 | 54.36% |

Supplemental Figure S2. PCA plot of (a) mRNA, (b) miRNA and (c) lncRNA expression based on RNA-seq
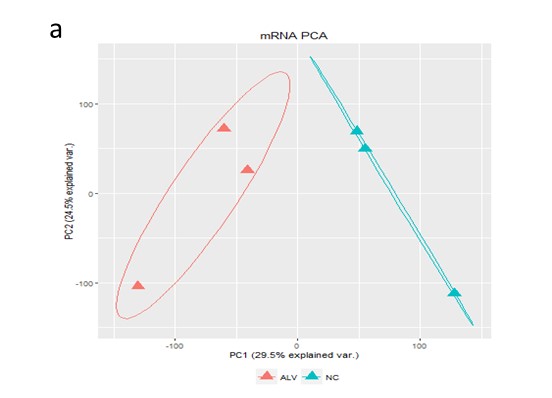
data


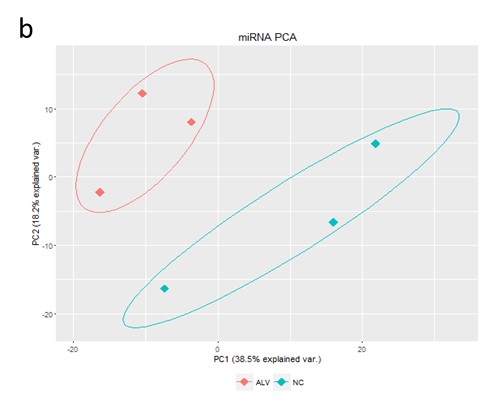

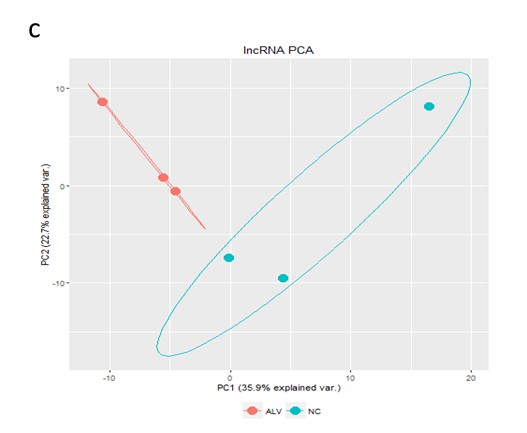


Supplemental Table S3. List of top 20 differentially expresses genes based on absolute log_2_ fold changes. Host defense peptide genes are highlighted in the down-regulated group, and immune related genes are highlighted in the up-regulated group.

| **Gene Name** | **Log_2_ FC** | **P-Value** | **FDR** | **Description** |
| --- | --- | --- | --- | --- |
| **GAL2** | **-3.40** | **3.79E-36** | **1.71E-32** | **Gallinacin-2** |
| **GAL1** | **-3.09** | **5.11E-30** | **8.65E-27** | **Gallinacin-1** |
| LECT2 | **-3.07** | 2.14E-31 | 4.83E-28 | myeloid protein 1 precursor |
| S100A12 | **-2.96** | 2.71E-28 | 4.08E-25 | S100 calcium binding protein A12 |
| **GAL6** | **-2.93** | **1.16E-20** | **7.16E-18** | **Gallinacin-6** |
| SERPINB10 | **-2.81** | 1.35E-26 | 1.66E-23 | Heterochromatin-associated protein MENT |
| TGM3 | **-2.76** | 1.18E-24 | 1.23E-21 | transglutaminase 3 |
| CALB1 | **-2.69** | 7.52E-24 | 6.47E-21 | Calbindin |
| **GAL7** | **-2.66** | **7.64E-24** | **6.47E-21** | **Gallinacin-7** |
| GFRA2 | **-2.60** | 1.3E-20 | 7.65E-18 | GDNF family receptor alpha-2 precursor |
| **GAL4** | **-2.49** | **1.72E-15** | **4.32E-13** | **Gallinacin-4** |
| GRPR | **-2.18** | 7.88E-15 | 1.75E-12 | gastrin-releasing peptide receptor |
| **CATHL2** | **-2.14** | **4.81E-15** | **1.1E-12** | **Cathelicidin-2** |
| CCR8L | **-2.02** | 6.62E-14 | 1.38E-11 | chemokine (C-C motif) receptor 8 |
| MMP13 | **-2.02** | 2.31E-15 | 5.69E-13 | matrix metallopeptidase 13 |
| **CATHL1** | **-1.97** | **3.55E-12** | **5.89E-10** | **Cathelicidin-1** |
| OLFM1 | **-1.91** | 2.01E-12 | 3.48E-10 | Noelin |
| RXFP1 | **-1.83** | 6.38E-13 | 1.2E-10 | relaxin/insulin-like family peptide receptor 1 |
| IFNA | **-1.79** | 9.4E-12 | 1.5E-09 | Interferon alpha |
| IL9R | **-1.76** | 6.74E-10 | 8.22E-08 | interleukin 9 receptor |
| UN | **4.12** | 1.94E-45 | 2.62E-41 | Uncharacterized protein |
| HBAD | **2.99** | 2.7E-30 | 5.22E-27 | Gallus gallus alpha-D-globin (HBAD) |
| PCP4 | **2.91** | 2.39E-09 | 2.68E-07 | Purkinje cell protein 4 |
| **Mx** | **2.84** | **1.35E-27** | **1.82E-24** | **interferon-induced GTP-binding protein Mx** |
| **IFN-γ** | **2.79** | **4.7E-20** | **2.65E-17** | **Interferon gamma** |
| GZMA | **2.69** | 3.27E-24 | 3.17E-21 | granzyme A precursor |
| **IFI27L2** | **2.65** | **8.72E-24** | **6.94E-21** | **interferon alpha-inducible protein 27-like protein 2** |
| LL | **2.58** | 3.91E-19 | 1.76E-16 | lung lectin precursor |
| SNORA5 | **2.50** | 2.42E-21 | 1.56E-18 | Small nucleolar RNA SNORA5 |
| DIO2 | **2.44** | 6.61E-17 | 1.95E-14 | deiodinase |
| HBG2 | **2.44** | 1.2E-21 | 8.58E-19 | Hemoglobin subunit beta |
| **CIDEA** | **2.44** | **1.51E-19** | **7.32E-17** | **cell death activator CIDE-A** |
| HBAA | **2.43** | 2.03E-21 | 1.37E-18 | Gallus gallus hemoglobin |
| **CCL4** | **2.43** | **7.25E-18** | **2.58E-15** | **chemokine-like ligand 1 precursor** |
| SPINK7 | **2.40** | 4.47E-19 | 1.95E-16 | Ovomucoid |
| NADP | **2.34** | 1.25E-18 | 4.97E-16 | NAD(P)(+)--arginine ADP-ribosyltransferase |
| SLC4A1 | **2.33** | 5.32E-17 | 1.6E-14 | solute carrier family 4 (anion exchanger) |
| RHAG | **2.24** | 6.03E-18 | 2.2E-15 | ammonium transporter Rh type A |
| U3 | **2.23** | 3.77E-18 | 1.42E-15 | Small nucleolar RNA U3 |
| CMPK2 | **2.23** | 2.4E-18 | 9.28E-16 | cytidine monophosphate (UMP-CMP) kinase 2 |

Supplemental Table S4. List of significant upstream regulators and target molecules in virus group.

Supplemental Table S5. List of significant upstream regulators and target molecules in miRNA group.

Supplemental Table S6. Forward and reverse primers used for qPCR validation of RNA-seq.

| **Gene** | **Forward Primer（5'- 3'）** | **Reverse Primer（5'- 3'）** | **Product Size（bp）** |
| --- | --- | --- | --- |
| **IL10**  **TNFRSF18**  **IL10R1**  **TLR4**  **IRF8**  **Ig** | ACCAGCACCAGTCATCAGCAG  AACCATCCCACCACAGAGAGG  TTCGCCATTGTCCTCTTCCTT  CCATCCCAACCCAACCACA  ACAGCAACAAGCAGGGCATC  ACCAACAGACCCTCGGACATC | TCCCGTTCTCATCCATCTTCTC  CTCGGAGGTTGGAAGTAGTAAGGT  TCCTGTTCCATCCAGAGTGAGC  CACCCACTGAGCAGCACCAA  CGAATATCTTCACCACCTCATCC  CGAACAGAAATAGACAGCCTCATC | 160  103  131  123  126  112 |
| **B2M** | TCACGCTGATGAAGGATGGC | CACTTGAAGACCTGCGGCTC | 175 |
| **IRF1**  **GAL6**  **GAL2**  **GAL1**  **GAL7**  **CATHL1**  **CATHL2**  **CAPN3**  **IL9R**  **IFN-γ**  **IL21**  **STAT1**  **IL21R**  **SLC11A1**  **IRG1**  **TNFSF8**  **TIFA**  **IRF10**  **IL18** | AGGATGCTCCCACCTCTGACAA  TACCTGCTGCTGTCTGTCCTCTT  GACGACTGCGACTTCAAGGAGA  ATGCGGATCGTGTACCTGCTC  CTGTCTGTCCTCTTTGTGGTGCTC  CTGCGAGTTCAAGGAGGACG  GACGACTGCGACTTCAAGGAGA  CGAAGTGACATTGGAGAACTGATT  CTGCGCTGAACAAGACACCAC  ATCATACTGAGCCAGATTGTTTCG  GGTGAAAGATAAGGATGTCGAATTG  GGAAACGGCTACATTAGGACTGA  TTGGACACTATTGACAGCGGC  GGAAACGGCTACATTAGGACTGA  GAACGCACTGGGTGGCTGAT  AGGGAGCTGCTGCATACATGA  TCTGAAGAATGCGGACAAGGA  CTGGACATCTCCGAGCCTTACA  ATTCAGCGTCCAGGTAGAAGATAAG | GGAGTGCTGGTTAGTCGTTCTGC  TAGTCCACTGCCACATGATCCA  ATCTTTCTCAGGAAGCGGCC  TTCAGAAATGCACAGAAGCCACT  CCAATCCAGTAATATGGCCTTCG  TTGTATCCTGCAATCACAGTCCTG  ATCTTTCTCAGGAAGCGGCC  TTGGCATTATGGTCCAGGCT  CTTTGAAATACTGTCGTCTGGCTC  TCAAGTCGTTCATCGGGAGC  CTTGGCTGTTTCTTGGCTGTAAT  CCATCCGAGATACCTCATCAAACT  CCTGCTCAAGAGGAACGGACT  CCATCCGAGATACCTCATCAAACT  TTCTGAGTTAGGGCTGGGTCTG  GGAACAGTTGGCAAAGTGGAA  ACTGTGAGCTGGACGGTGGA  TGTGGGTCCTTACTGCTTGTGG  CACCAGGAATGTCTTTGGGAAC | 140  159  158  125  131  165  158  121  169  178  122  121  109  121  149  173  169  113  110 |

Supplemental Figure S7. Correlation between RNA-seq and Fluidigm qPCR.


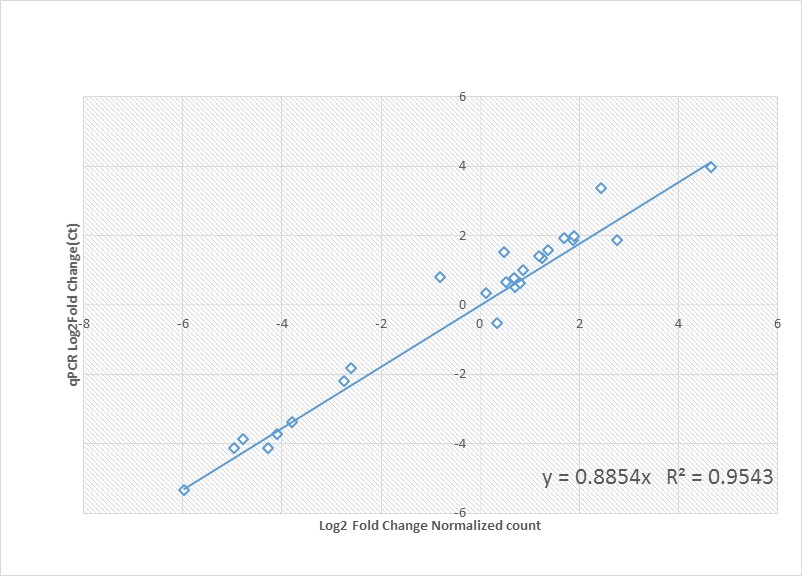


Supplemental Figure S8. Physical characteristics for host response to ALV challenge. Immune index =immune organ weight / body weight * 100. (a) The ratio of Immune index (ALV group/Control group) up to 40 days post-injection for each tissue. (b) Overall body weight, and (c) Splenic weight. (d) Infection rate of ALV challenged chickens.


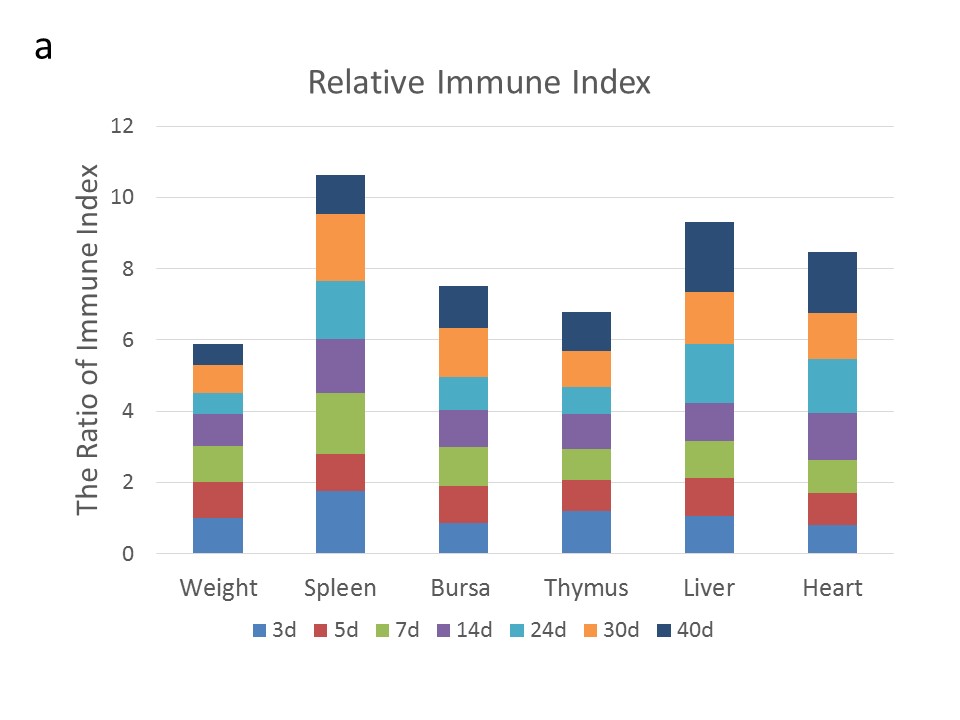

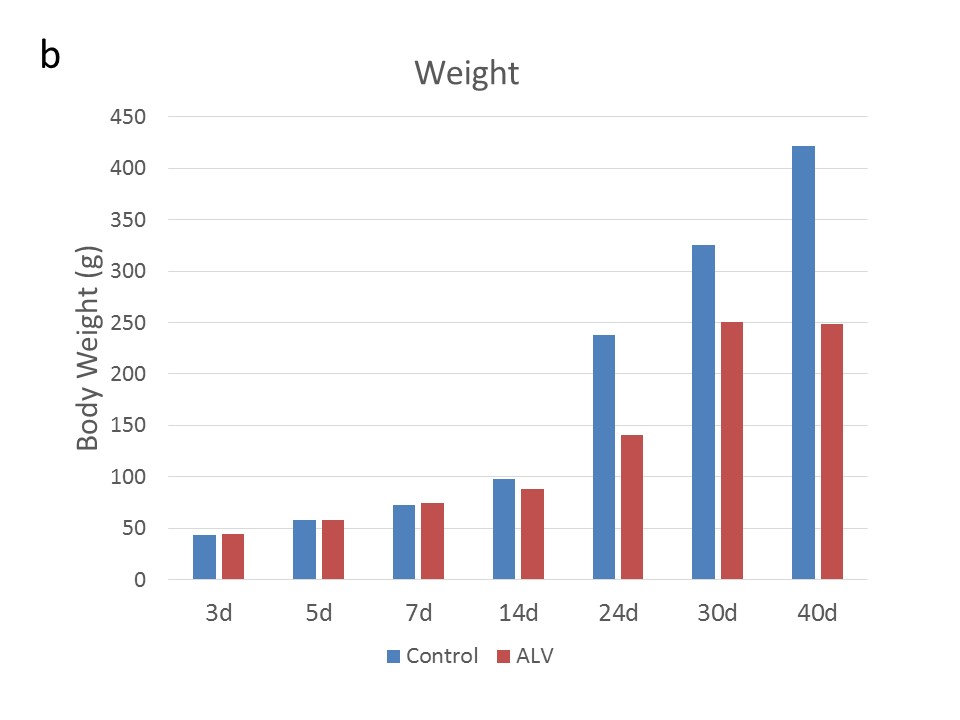


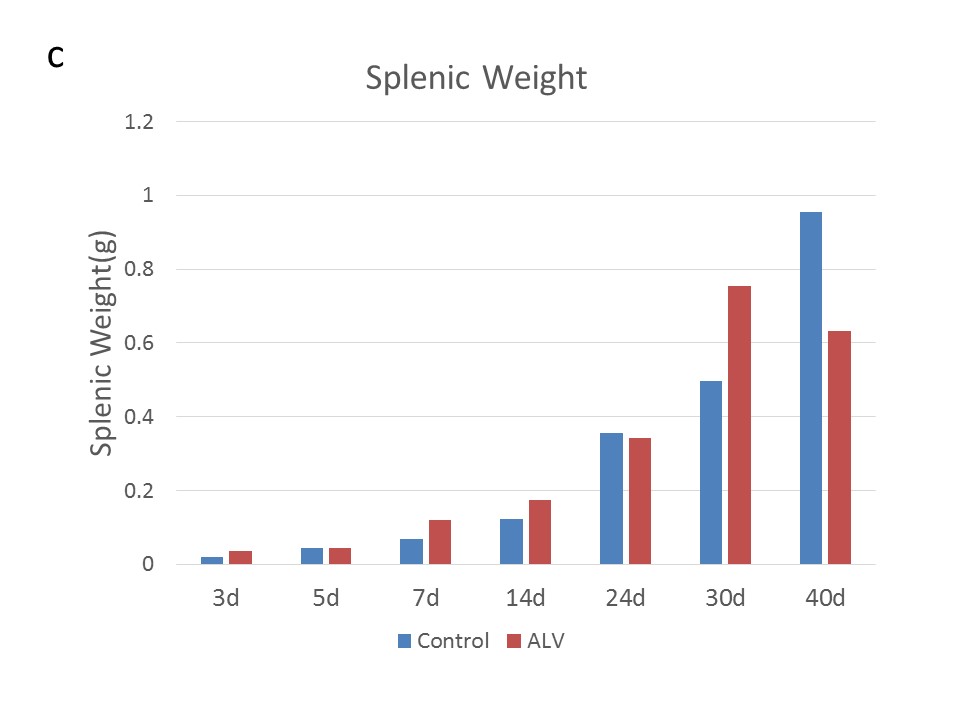

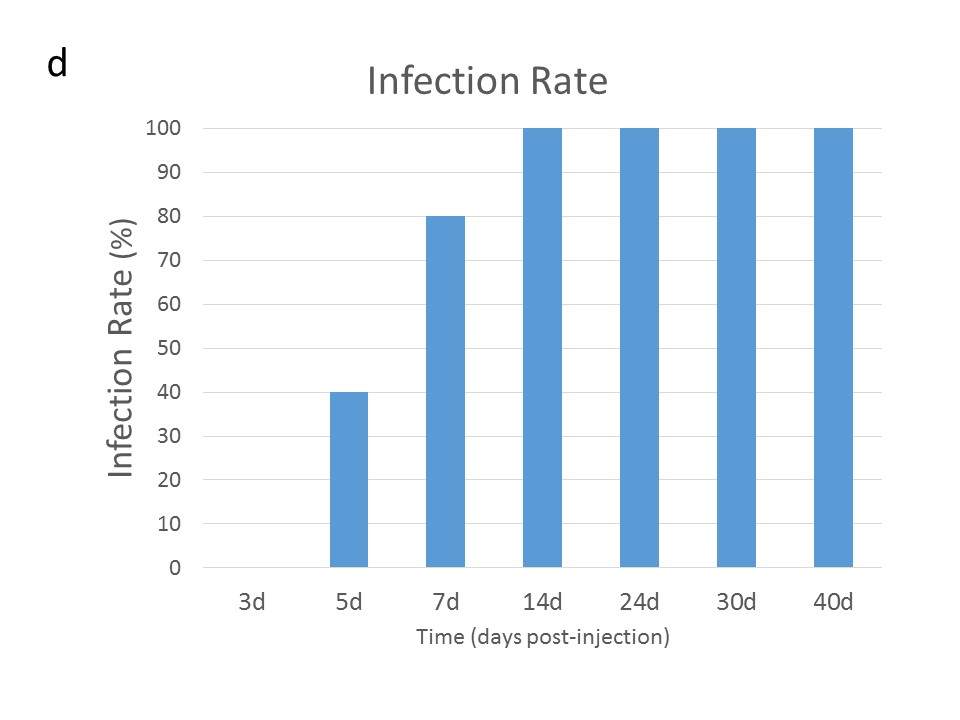


Supplemental Figure S9. Experiment and analysis design.


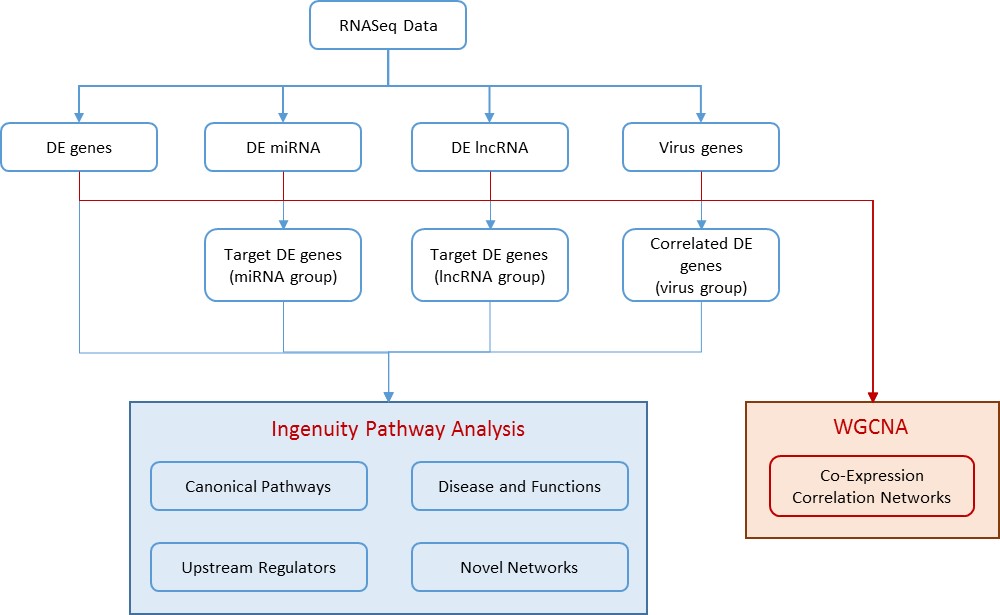

Supplement: Supplementary Information [file srep46156-s1.doc]
